# EHPnet: *EHP* International Program

**Published:** 2008-07

**Authors:** Erin E. Dooley

http://www.ehponline.org/international/

Through its International Program *EHP* seeks to expand the quality of and access to scientific information around the world.With its new International Program website, *EHP* makes it easy for readers to learn about the journal’s international outreach activities and to access the translated content.

The website contains four main sections. Global Access describes *EHP*’s provision of complimentary print subscriptions to institutions in developing nations, its participation in the eGranary Digital Library and JSTOR online repositories, and its adherence to an Open Access publishing model. The section on Capacity Building describes the journal’s work to assist scholarly journals in developing countries (currently working with Mali, Chile, and Brazil) in overcoming obstacles to publication. Closely related to this section is a third area, Journal Partnerships, which describes *EHP*’s program of fostering scientific exchange through wider dissemination of scientific information to broader audiences.

Translated Content offers easy access to the various forums in which *EHP* content is provided in languages other than English. Since 2001, a Chinese-language edition of *EHP* has been published quarterly in partnership with the Shanghai Municipal Center for Disease Control and Prevention. Selected journal content is also available in Portuguese in the Brazilian public health journal *Ciência & Saúde Coletiva* and in Spanish in the Chilean occupational and environmental health publication *Ciencia y Trabajo*. A Palestinian medical journal, *Annals of Alquds Medicine*, also has recently begun translating selected *EHP* news articles into Arabic.

